# Discovery of cryptic plant diversity on the rooftops of the Alps

**DOI:** 10.1038/s41598-021-90612-w

**Published:** 2021-05-27

**Authors:** Florian C. Boucher, Cédric Dentant, Sébastien Ibanez, Thibaut Capblancq, Martí Boleda, Louise Boulangeat, Jan Smyčka, Cristina Roquet, Sébastien Lavergne

**Affiliations:** 1 Univ. Grenoble Alpes, Univ. Savoie Mont Blanc, CNRS, LECA, 38000 Grenoble, France; 2 Parc National Des Écrins, Domaine de Charance, Gap, France; 3 Univ. Grenoble Alpes, CNRS, Sciences Po Grenoble, Pacte, 38000 Grenoble, France; 4 Department of Plant Biology, University of Vermont, Burlington, VT 05405 USA; 5 Center for Theoretical Study, Charles University and the Academy of Sciences of the Czech Republic, 110 00 Prague, Czech Republic; 6 Systematics and Evolution of Vascular Plants (UAB) - Associated Unit To CSIC, Departament de Biologia Animal, Biologia Vegetal I Ecologia, Facultat de Biociències, Universitat Autònoma de Barcelona, 08193 Bellaterra, Spain

**Keywords:** Phylogenetics, Speciation, Taxonomy

## Abstract

High elevation temperate mountains have long been considered species poor owing to high extinction or low speciation rates during the Pleistocene. We performed a phylogenetic and population genomic investigation of an emblematic high-elevation plant clade (*Androsace* sect. *Aretia,* 31 currently recognized species), based on plant surveys conducted during alpinism expeditions. We inferred that this clade originated in the Miocene and continued diversifying through Pleistocene glaciations, and discovered three novel species of *Androsace* dwelling on different bedrock types on the rooftops of the Alps. This highlights that temperate high mountains have been cradles of plant diversity even during the Pleistocene, with in-situ speciation driven by the combined action of geography and geology. Our findings have an unexpected historical relevance: H.-B. de Saussure likely observed one of these species during his 1788 expedition to the Mont Blanc and we describe it here, over two hundred years after its first sighting.

## Introduction

Documenting incipient or recent events of speciation is of paramount importance to understand the mechanisms generating biodiversity, but also to implement adequate conservation actions based on correct species delimitation. This stake is prominent for high-elevation ecosystems (i.e., those from the upper alpine and nival vegetation belts, typically above 2500 m a.s.l. in the European Alps^1^), whose biodiversity appears to be disproportionately threatened by ongoing climate change^2^. In contrast to mountainous environments in general^3,4^, high-elevation ecosystems are species poor due to extremely harsh environmental conditions^5,6^. The fact that high-elevation environments are biological deserts is generally explained by the combination of high extinction rates during Pleistocene glaciations^7,8^ and low speciation rates caused by low ecosystem productivity^9^. In spite of their low diversity, these environments harbor a high proportion of endemics and retain an important conservation value^5,10^.

In this article we focus on the European Alps (hereafter, ‘the Alps’) and posit the existence of yet undescribed cryptic plant species within high-elevation ecosystems. Such cryptic diversity is probably due to the nature of the main processes responsible for speciation in the alpine flora: allopatric isolation among mountain ranges and adaptation to divergent substrates^7,8,11,12^, which normally leave little imprint on species’ morphology. Several reasons explain why this cryptic diversity might lay unrecognized: (1) Alpine plant taxonomy has mostly been studied using morphology, which may dramatically underestimate diversity^13^; (2) phylogenetic studies of Alpine plants have mostly relied on limited sampling within species and limited sequencing effort^14^, reducing our ability to identify recently diverged lineages; and (3) high alpine environments have long remained unexplored due to their difficult access. Taking advantage of improvements on all those aspects, recent plant surveys suggest that diversity in these environments is much higher than previously assumed^15^. Improving species delimitation and identification of cryptic species is crucial for a better biodiversity assessment on rooftops of the Alps and to inform conservation strategies for these ecosystems, as done elsewhere and for other organisms^16,17^. From a broader perspective, accurate species delimitations are also key for testing theories in biogeography^18,19^ or to improve our understanding of the speciation process^20,21^.

Here we study the genus *Androsace* (Primulaceae), which contains some of the vascular plant species dwelling at the highest elevations and the coldest places on Earth^22,23^. We focus on one part of the genus, *Androsace* section *Aretia,* which has its center of diversity in the Alps but is distributed in all European mountains, with some additional species in North America^24,25^. The section has diversified in the last 15 Myr, largely thanks to the emergence of a key innovation, the cushion life form^26^, that enabled some of its species to conquer the highest elevations^25,27^. While understanding the speciation process was our initial incentive to study *Androsace* sect. *Aretia*, we were also intrigued by the recent description of a putative new species in the Mont Blanc range^28^. A subsequent taxonomic revision then raised the suspicion that other species may remain to be described in this group^29^, a highly puzzling fact after almost 300 years of study of the Alpine flora since von Haller^30^. Relying on extensive sampling, especially in the Western Alps, and on thousands of SNPs obtained through double digest restriction site associated DNA sequencing^31^, we conducted a series of phylogenomic and population genomic analyses aimed at: (1) providing a robust species-level phylogeny for *Androsace* section *Aretia* and (2) testing the status of three putative new species. Genomic evidence was later corroborated using species’ bedrock affinity and morphology, contributing to a better understanding of speciation mechanisms and diversification dynamics in the high-elevation flora of the European Alps.

## Results

**Figure 1:**
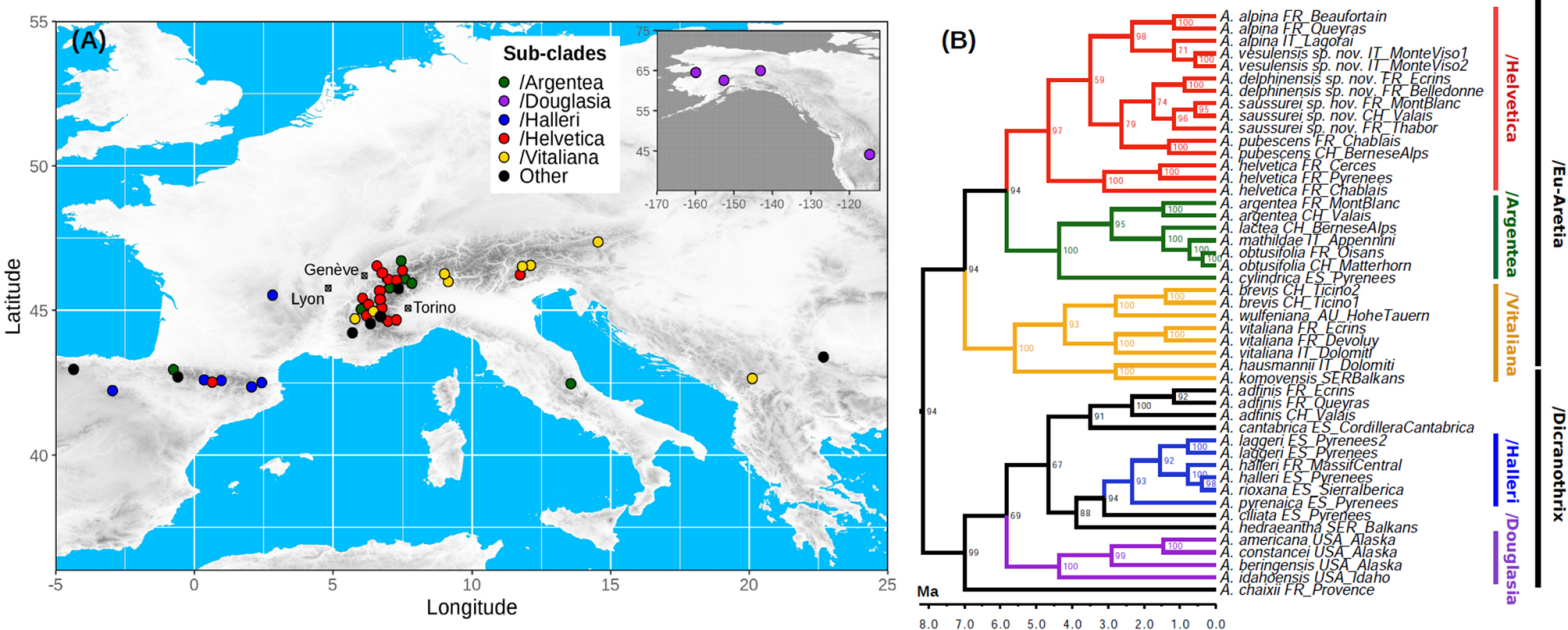
(**A**) Geographic distribution of samples used for the phylogenomic analysis of *Androsace* sect. *Aretia*. The background map shows elevation in shades of grey and was drawn using the R package *raster*^62^. (**B**) Maximum-likelihood phylogeny of *Androsace* sect. *Aretia* based on the concatenation of 2700 ddRAD tags together totaling 314,363 bp, and dated using penalized likelihood with secondary calibration. Bootstrap support is displayed at nodes and time is shown on the x axis in million years. The figure was drawn using the program FigTree (http://tree.bio.ed.ac.uk/software/figtree/).

We used a large genomic dataset to improve our understanding of the systematics of *Androsace* sect. *Aretia.* We generated ddRAD-seq^31^ data for 88 individuals spanning the whole distribution of the clade (Fig. 1A) and 28 out of its 33 currently recognized species (85%), including all European ones (24 spp.). While most species were represented by one to three accessions (see Table S1), we conducted a much denser sampling for a small clade of high-elevation cushion species suspected to include cryptic taxa that was achieved through decade-spanning alpinism expeditions on most of the highest summits of the Western Alps. The resulting ddRAD tags were aligned to the reference genome of another Primulaceae species, *Primula veris* L.^32^. This allowed to infer a high-quality phylogeny of *Androsace* sect. *Aretia* using both maximum-likelihood (hereafter, ‘ML’) on the concatenation of all ddRAD tags (314,363 bp) and species tree inference using unlinked polymorphic sites only (2461 SNPs). Both approaches were largely congruent and led to a highly supported phylogenetic hypothesis (Figs. 1B, S2, S3, SI Sect. 2.1). They supported the split of sect. *Aretia* into two large clades. The first one included North-American and European species, mostly specialists of subalpine and alpine habitats (clade /Dicranothrix s*ensu*^24^)⁠, whereas the second one mainly consisted of cushion species from the Alps and adjacent mountain ranges that grow at the highest elevations and frequently occur in the nival zone (clade /Eu-Aretia s*ensu*^24^)⁠. Furthermore, both ML and species tree inference supported the existence of the same five subclades. One of them, /Douglasia, corresponds to the former genus *Douglasia* Lindley and the four others were named after their most widespread species: /Argentea, /Halleri, /Helvetica and /Vitaliana. All of these clades contain almost exclusively cushion species, except for species of the /Halleri clade, which are perennial rosettes. Divergence time estimation performed with penalized-likelihood on the ML phylogeny and calibrated with ages derived from a previous study of Primulaceae (*i.e.,* secondary calibration) suggested that all five subclades might have originated before the Pleistocene, possibly in the Pliocene (Fig. 1B, Fig. S2, SI Sect. 2.2).

**Figure 2:**
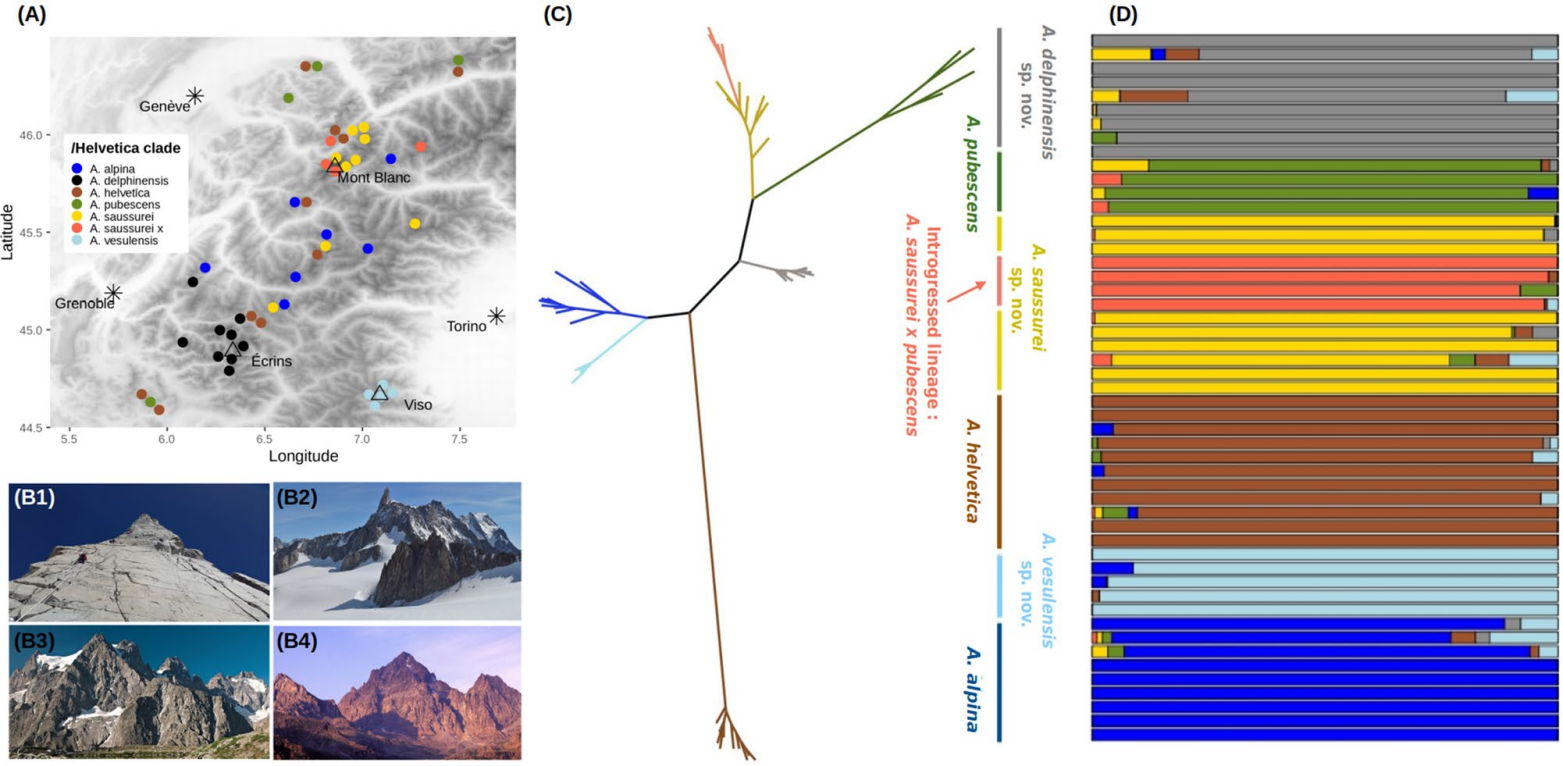
Genetic structure within the /Helvetica clade. (**A**) Distribution of study samples within the western Alps, spanning the three formerly described species and the three new species described in this study. Individuals of *A. saussurei* introgressed by *A. pubescens* are labeled as ‘*A. saussurei* x’. The background map shows elevation and was drawn using the R package *raster*^62^. (**B**) Pictures depicting the typical high elevation cliff habitats that have been explored for the present study (**B1**), and the three main mountain ranges where putative novel species occur, namely Mont Blanc (**B2**), Ecrins (**B3**), and Monte Viso (**B4**). (**C**) Phylogenetic relationships between the 51 individuals of /Helvetica, inferred using ML on a concatenation of 23,780 *loci* (276,745 bp). The figure was drawn using the program FigTree (http://tree.bio.ed.ac.uk/software/figtree/). (**D**) Assignment of the same individuals to seven genetic clusters^33^ as identified based on a strict selection of 381 unlinked SNPs.

Based on a denser sampling (Fig. 2A), we then focused on the /Helvetica clade, which was estimated to have originated at 5.8 Ma (95% HPD: 2.5–8.3 Ma). This clade contains three currently recognized cushion species, all of which grow at high elevations, *A. alpina*, *A. helvetica*, and *A. pubescens*, as well as putative new taxa, which is the reason why we investigated its systematics further. We first used a constrained clustering method^33^ to delimit genetic groups without a priori taxonomic assignment. Results supported an optimal number of K = 7 clusters, which almost perfectly aligned with the phylogeny of these 51 individuals (Fig. 2C,D). All individuals of *A. helvetica* were assigned to the same cluster. In contrast, individuals morphologically assigned to *A. alpina* were split into two distinct clusters (Fig. 2D): one comprising individuals growing on ophiolites of Monte Viso (hereafter, *A. vesulensis* sp. nov.) and another one comprising all other individuals (hereafter, *A. alpina*). Individuals morphologically assigned to *A. pubescens* were split into three clusters (Fig. 2D): one including all individuals growing on limestone (hereafter, *A. pubescens*), one including individuals growing on siliceous substrates in the Mont Blanc and neighboring ranges (hereafter, *A. saussurei* sp. nov.) and the last one including individuals growing on siliceous substrates in the Central French Alps (hereafter, *A. delphinensis* sp. nov.). Finally, further analyses showed that the last cluster (Fig. 2D), which comprised four individuals with an intriguing morphology, resulted from introgression between *A. pubescens* and *A. saussurei* sp. nov. (SI Sect. 3.2).

The taxonomic status of the genetic clusters that did not align with currently recognized species (i.e., *A. saussurei* sp. nov.*, A. delphinensis* sp. nov. and *A. vesulensis* sp. nov.) was tested using multiple lines of evidence. We first conducted molecular species delimitation, which identifies species as independent evolutionary lineages that do not exchange genes anymore⁠^34,35^. Bayes Factor Delimitation^36^ indicated decisive support for a scenario in which *A. alpina* and *A. vesulensis* sp. nov. would be considered different species rather than forming a single one (Bayes Factor, hereafter BF = 1052—support for a given scenario is considered decisive when for BF > 150, Table S2). Using the same approach, we also found decisive support for considering *A. pubescens, A. saussurei* sp. nov. and *A. delphinensis* sp. nov. as distinct species, rather than lumping *A. saussurei* sp. nov. and *A. delphinensis* sp. nov. together (BF = 690, Table S2), or even lumping the three clusters into a single species, as assumed by current taxonomy (BF = 1698). Genetic PCAs confirmed that the three new species are discrete genetic groups and not arbitrary portions of a larger genomic cline (SI Sect. 3.3, Figs. S11–S12). F_ST_ between newly described species and their close relatives range from 0.08 to 0.3, and are thus of the same order of magnitude as the ones measured between recognized species like *A. alpina* and *A. pubescens* (F_ST_ = 0.12) or *A. pubescens* and *A. helvetica* (F_ST_ = 0.17, SI Sect. 3.3). In addition, mantel correlograms show that the genetic structure that we interpret as evidence for two distinct species is not merely due to isolation by distance (Fig. S13). While a hybrid origin of *A. saussurei* sp. nov. and *A. delphinensis* sp. nov. was ruled out by Approximate Bayesian Computation analyses^37^, we cannot exclude the possibility that *A. vesulensis* sp. nov. emerged as a hybrid species between *A. alpina* and *A. pubescens* (SI Sect. 3.4).

In addition to molecular delimitation, we used ecological data to confirm the status of these putative species. Based on our extensive prospecting we first refined the chorology and bedrock preferences of these new species and found that they have largely allopatric distributions compared to their close relatives (Fig. 2A, Fig. S16). Contrary to the closely related *A. alpina*, which grows on schist or other siliceous rock screes, *A. vesulensis* sp. nov. grows on more stable rock crevices or cliffs, always on ophiolites. Our new circumscription of *A. pubescens* restricts this species to limestone crevices or cliffs (contrary to previous descriptions which treated it as a bedrock generalist), while the closely related *A. saussurei* sp. nov. and *A. delphinensis* sp. nov. also grow on rock crevices or cliffs, but exclusively on siliceous bedrocks (e.g., granite, quartzite, gneiss, or sandstone). These geographic and edaphic differences add support to give species rank to each of the three taxa newly described in this study.

**Figure 3:**
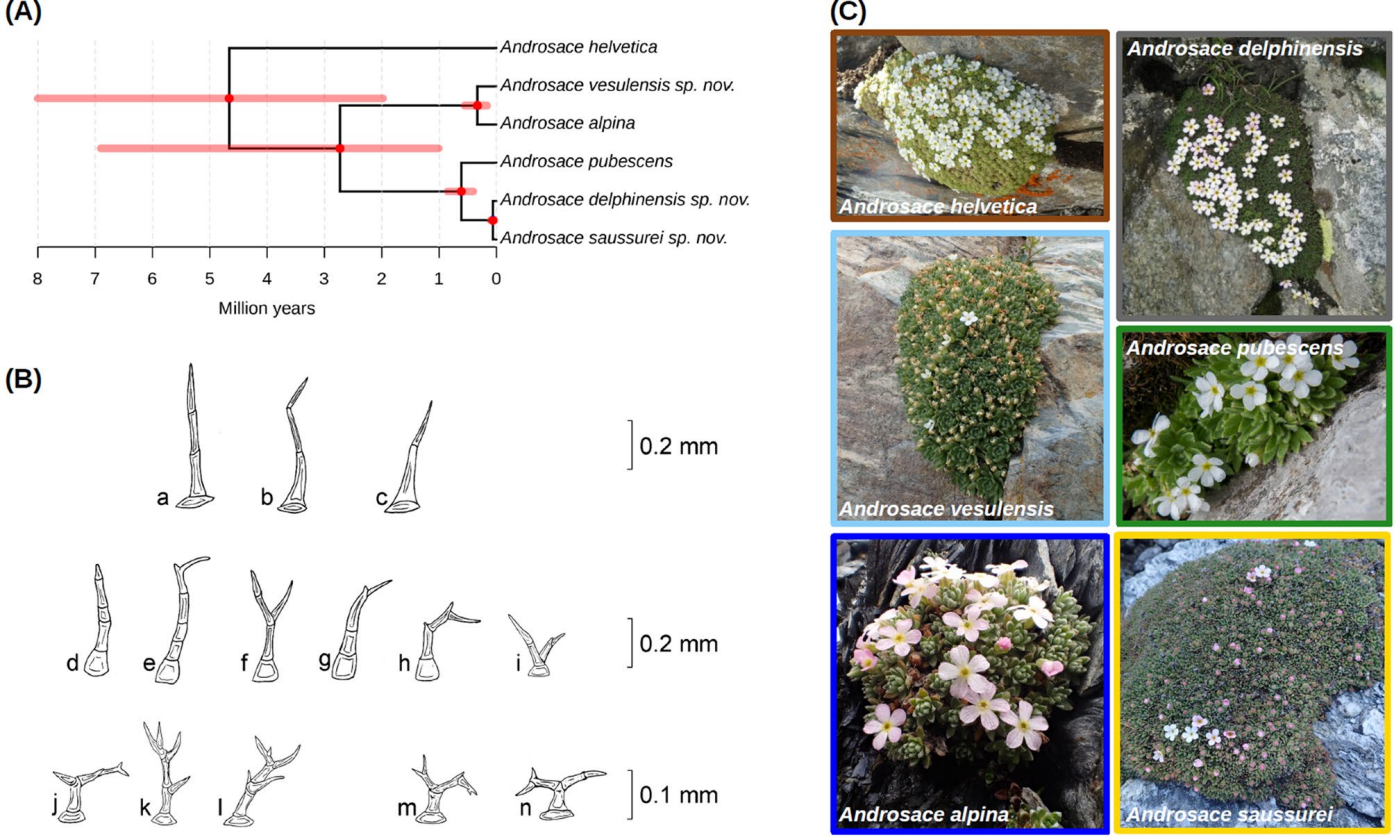
Genetic and morphological delimitations of species within the /Helvetica clade. (**A**) Species tree inferred from a strict selection of 381 unlinked SNPs, all nodes received a posterior probability of 1.00 except for the most basal one which was supported with 0.45 posterior probability. The figure was drawn using the *ggtree* R package (http://bioconductor.org/packages/release/bioc/html/ggtree.html). (**B**) Drawings of the different trichome morphologies characterizing species of the /Helvetica clade. (**a**–**c**) *A. pubescens* and *A. helvetica*; (**d**–**i**) *A. saussurei* sp. nov. and *A. delphinensis* sp. nov. (**d**,**e**,**g**) leaves only; (**i**) peduncles only; (**f**,**h**) both leaves and peduncules); (**j**–**l**) *A. vesulensis* sp. nov.; (**m**–**n**) *A. alpina*. All drawings from C. Dentant (**C**) Pictures depicting the overall morphology of different species delimited within the /Helvetica clade. Pictures from L. Boulangeat, S. Ibanez, and S. Lavergne.

Voucher samples were later examined to look for morphological criteria that could serve for species diagnosis. We found only subtle differences, mostly in the morphology of leaves and peduncles trichomes, which are described in the taxonomic treatment below (see also Fig. 3B). These morphological differences themselves do not allow distinguishing all six species, but when combined to geographic and edaphic information they can serve to do so (see *Determination key* in SI Sect. 5.). Finally, we inferred a species tree for the six newly circumscribed species of /Helvetica using the program SNAPP^38^. All phylogenetic relationships were strongly supported except for the most basal divergence between *A. helvetica* and the rest of the clade (Fig. 3A, SI Sect. 3.5). Using a prior on the crown age of /Helvetica derived from the dated phylogeny of *Androsace* sect. *Aretia* obtained above, we found that the three newly described species likely originated within the last million years (Fig. S15). These multiple lines of evidence lead us to recognize three new species in *Androsace* sect. *Aretia*: *A. delphinensis*,* A. saussurei* and *A. vesulensis*, which we typify and formally describe at the end of the manuscript.

## Discussion

New species are still being frequently described from under-explored regions or under-studied taxonomic groups but it is extremely rare to discover novel angiosperm species in a region with a long floristic tradition, such as the European Alps. Against all odds, here we describe three new species of *Androsace* that grow on three of the most emblematic mountain ranges of the Alps (Fig. 2B): the one that Romans believed was the highest of the world during antiquity, Monte Viso (3841 m a.s.l.); one of the wildest ones, the Écrins (4102 m a.s.l.); and the rooftop of Europe, Mont Blanc (4810 m a.s.l.). Tribute must be paid to H.-B. de Saussure, who in 1788, during a journey on the Glacier du Géant (3350 m a.s.l, Mont Blanc range), found only one flowering plant species “*sometimes white, sometimes purplish*”^39^ and made the first observation of what we just described as *Androsace saussurei* sp. nov., more than two hundred years later. Another new species of *Androsace* with a rather similar morphology and partially overlapping geographic range was recently described (*A. albimontana* D. Jord. & Jacquemoud^40^). This species may be synonym to *A. saussurei* sp. nov., although the description of its morphology and habitat differ substantially from ours. Given that the covid-19 pandemic impeded us from reviewing the type of this taxon and in the absence of genetic study, it is unfortunately impossible to assess synonymy between the two species at this stage.

An important result of our study is that these new species are cryptic (Fig. 3C), which might explain why they had been overlooked until now. We did find some subtle morphological differences between species, though, as highlighted above and summarized in the taxonomic treatment. As we describe in alpine *Androsace* here, and as found earlier in arctic species of the genus *Draba*^13^, cryptic species may have recently arisen within arctico-alpine floras. While other studies of rapid species diversification have often highlighted morphological diversification^41,42^, our study adds to mounting evidence that rapid species diversification can also be decoupled from morphological differentiation^43,44^. The *Androsace* species we describe here perhaps result from rather ephemeral speciation processes^45^ due to Pleistocene climatic fluctuations that have generated morphologically cryptic but genetically distinct lineages. Our results thus suggest that plant species diversity may have been underestimated in high-elevation ecosystems, in particular nival ones, due to limited sequencing and sampling effort of plants dwelling in extreme high alpine environments.

Our study first provides an improved understanding of phylogenetic relationships in *Androsace* sect. *Aretia*. Using thousands of genetic *loci* obtained through ddRAD sequencing along with multiple accessions for the majority of species and mapping these *loci* to a reference genome yields a major improvement in our understanding of the evolutionary history of the whole *Androsace* sect. *Aretia*. It first establishes that geographic structure is marked within the section (Fig. 1), with one clade of North-American species (clade /Douglasia), another one largely confined to Western Europe (clade /Halleri), a clade widespread in European high mountains (clade /Vitaliana), and two clades with most of their diversity in the Central and Western Alps (clades /Argentea and /Helvetica). Species bearing the cushion life form, an adaptation to cold and dry environments that has appeared more than a hundred times across angiosperms^46^, are found in all major subclades of *Androsace* sect. *Aretia*. Finally, we find that all subclades cited above originated during the Pliocene and continued diversifying throughout the Pleistocene, a period marked by accelerated erosion rates due to global cooling and later glaciations which led to an increase in relief^47,48^. The formation of deep valleys separating the habitats occupied by species of section *Aretia* might thus have favored its diversification. The precise dating of these events should be taken cautiously since it relies on secondary calibration, but, if anything, these ages are overestimated given that the original study had obtained rather old divergence times compared to other studies of Primulaceae^7^.

We provide evidence for recent plant species origination in some of the most extreme environments on Earth. High-elevation habitats in the Alps host relatively little plant life^5,22^ and have thus been long viewed as historical sinks of diversity^7–9^, due to low speciation owing to harsh climatic conditions and to high extinction driven by Pleistocene glaciations. Instead, we show that the number of species recognized in the /Helvetica clade should be raised from three to six, and that three speciation events likely took place during the Pleistocene, a period during which the Alps were largely covered by glaciers. The occurrence of Pleistocene speciation, together with the distribution of species in the interior of the Alps (Fig. 1), suggests that plants of the /Helvetica clade may have survived in nunataks located above glaciers and diversified during glacial periods^49^. This is a plausible scenario given that these plants frequently grow on high-elevation cliffs above glaciers today. For species of /Helvetica it has even been suggested that these refugia might have been located both in the center and at the periphery of the Alps^50^. More generally, the origin of major subclades within *Androsace* sect. *Aretia* in the Pliocene and the occurrence of several speciation events during the Pleistocene supports the idea that mountain floras worldwide are the result of fast and recent evolutionary radiations^51^.

Our work employs the unified species concept of de Queiroz, which identifies species as independently evolving metapopulation-level lineages^34^. The operational criteria that we use to delimit species include three sources of evidence: genomic divergence (coming from both phylogenetic and genetic clustering analyses), morphological differentiation (although subtle), and ecological differentiation (mainly through bedrock affinities). Our approach thus embraces the emerging paradigm of integrative taxonomy^52,53^. The new species described in this study have allopatric distributions with their closest relatives (Fig. 2). This supports the prominent role of allopatric speciation in the flora of the European Alpine System, which can be conceived as an archipelago of ‘sky islands’ among which gene flow is probably very limited^7,8,54^. But the other striking observation is that these new species grow on distinct substrates compared to their close relatives. While *A. pubescens* exclusively grows on limestone, its sister clade, which is formed of *A. saussurei* sp. nov. and *A. delphinensis* sp. nov. is found on siliceous rocks. Similarly, the widespread *A. alpina* is restricted to siliceous rocks whereas its sister species *A. vesulensis* grows on mafic or ultramafic ophiolites. Substrate is known to have played an important role in the phylogeography of the Alpine flora^55^ and has also been proposed as a driver of speciation^7,11,12^. This is likely because of the trade-offs required for adaptation to alternative soil chemistries, but also because bedrock type is typically uniform across spatial scales of a few kilometers in the Alps and because it has remained constant during the whole geological history of the Alps, except for the erosion of some sedimentary rocks capping nowadays exposed igneous ones^56^. This illustrates that allopatric and ecological speciation are intertwined rather than mutually-exclusive mechanisms. In contrast, climate doesn’t seem to be an axis of niche divergence in the Alpine flora^7^, probably because it varies over short distances along elevational gradients and because it has varied drastically over timescales of a few thousand years following glacial cycles. In /Helvetica, the spatial separation and temporal constancy of bedrock types would have triggered ecological speciation, leaving enough time for different plant lineages to adapt to different substrates, while the same may not have been possible for climate. Plants of the /Helvetica clade would be especially prone to substrate specialization given that they live on cracks or screes of the bedrock and are thus directly affected by its properties. Importantly, reproductive isolation does not seem to be complete between species. This is suggested by the existence of hybrids between *A. pubescens* and *A. helvetica* previously found in the wild^50^ and the presence of individuals of *A. saussurei* sp. nov. introgressed by *A. pubescens* documented in this study. Furthermore, the possibility that *A. vesulensis* sp. nov. originated through hybrid speciation, even though it deserves further investigation, would also support the incompleteness of reproductive barriers in /Helvetica. This could be the result of allopatric divergence, during which intrinsic reproductive barriers are not selected and thus evolve rather slowly^57^. Field-based measures of gene flow between these taxa would be needed to determine the strength of contemporary reproductive barriers.

This systematic study of one of the plant clades found in the coldest environments on Earth bears importance for implementing appropriate conservation strategies in ecosystems at stake with the issue of climate change. We just unraveled the existence of cryptic species of high elevation *Androsace* using molecular data, but further scrutiny of fine morphological characters allowed proposing diagnostic traits for these new species. It has to be acknowledged that further investigation is needed to study trait differentiation between these taxa while accounting for the full range of variation of these traits. Despite the seemingly good news of this finding, the newly identified *A. delphinensis* sp. nov.*, A. saussurei* sp. nov.*,* and *A. vesulensis* sp. nov. are already facing important threats. Until a proper threat assessment is performed, these novel species inherit the protection status of the species they were previously included in (that is, *A. pubescens* and *A. alpina*) and are thus automatically protected in France, Italy, and Switzerland. But, given their restricted range sizes (Fig. 2) and the important risk they may face from climate change, either directly^2^ or possibly due to increased competition with colonizers from lower elevations^58^, there is little doubt that these novel species will require species-specific protection plans. Here we have only investigated the systematics of one clade, but high-elevation ecosystems probably harbor more unknown species that may disappear before ever being described. It is thus time to change our perception of these ecosystems, which are much more deserts of knowledge than deserts of life.

## Methods

Samples used in this study were collected during a decade of botanical expeditions spanning large elevation gradients on over 90 of the highest summits and passes of the Western Alps, most of the time requiring the means of alpinism techniques. For most of these high elevation sites, no botanical data had ever been recorded. This study used 88 individuals sampled throughout European mountains (Fig. 1A), including 1–3 individuals of all 24 European species of *Androsace* sect. *Aretia*, and a denser coverage of *A. alpina* (L.) Lam. (14 individuals, including one suspected new taxon), *A. helvetica* (L.) All. (11 individuals), and *A. pubescens* DC. (26 individuals, including two suspected new taxa). We also included one sample of four of the nine North-American species of the section, plus three outgroups (Table S1).

Total DNA from all samples was extracted using a DNeasy Plant Mini Kit (Quiagen, Germany). A double digest estriction site associated DNA experiment (hereafter, ddRAD-seq) was conducted using a modified version of the original protocol (^31^, see SI Sect. 1.1 for details) with PstI and MspI as restriction enzymes. The four libraries that we generated were sequenced on half a lane of Illumina Hi-Seq 2500 2 × 125 (Fasteris SA, Switzerland), generating more than 275 million reads of 2 × 125 bp. Rather than relying on de novo assembly of these loci, we preferred to align them on the reference genome of another species from the Primulaceae family: *Primula veris* L.^32^ and then call SNPs. This was done using the program ipyrad (https://github.com/dereneaton/ipyrad). We however verified that a dataset containing both loci aligned to the reference genome and de novo assembly of loci that did not align to the reference genome provided qualitatively similar results (see below). We also checked that technical replicates of our four ddRAD-seq libraries and sequencing runs produced comparable data prior to combining them for further analyses (Fig. S1). While some phylogenetic analyses used either all DNA base pairs sequenced or all SNPs that were called, for some other analyses we selected unlinked SNPs that were either located on different contigs of the reference genome or located on the same contig but > 10,000 bp apart (see details below and in SI).

Phylogenetic relationships within *Androsace* sect. *Aretia* were inferred using two different approaches: ML inference of all ddRADseq tags concatenated (314,363 bp) using IQ-TREE^59^ and species tree inference from 2461 unlinked SNPs using SVDquartets^60^. In both cases, clade support was measured using bootstrap and trees were rooted thanks to the inclusion of three outgroup taxa from the Primulaceae family, located at increasing phylogenetic distances from our ingroup: *A. septentrionalis* L., *Primula hirsuta* All., and *Lysimachia nummularia* L. (SI Sect. 2.1). We then dated the phylogram obtained from concatenation using penalized likelihood^61^. We calibrated two nodes of the phylogeny using median ages estimates from a previous study of Primulaceae^7^: the crown node of *Androsace* sect. *Aretia* (8.16 Ma) and the divergence between the genera *Androsace* and *Primula* (33.3 Ma). In an effort to reduce computing time, we only retained two to three individuals for each species of the /Helvetica clade for these phylogenetic analyses. These individuals were chosen as to maximize coverage of each species’ geographic range.

In order to revise the systematics of /Helvetica and to test the putative species status of newly discovered taxa, we used an integrative taxonomic approach combining genomics, geography, and habitat characterization. We started by doing a different SNP calling for the 51 individuals of the /Helvetica clade using the same pipeline as described above, which resulted in 23,780 loci aligned to the reference genome, containing a total of 276,745 bp and 7806 SNPs. In order to use population genetic measures on this recently diverged clade, we strictly filtered this initial SNP dataset: we only kept biallelic SNPs that had less than 40% missing data, that had minor allele frequencies > 4%, and that were unlinked (i.e., located on different contigs of the reference genome or > 10,000 bp apart on the same contig), yielding a final dataset of 381 SNPs. This number is rather low but we preferred working with these high-quality SNPs only rather than relying on de novo assembly of ddRADseq tags, which gave similar results (SI Sects. 3.1 and 3.2, Figs. S5 and S6, Figs. S8 and S9). Genetic clusters were then inferred with no a priori grouping using the sNMF algorithm, which aims at estimating individual ancestry^33^. We tested numbers of genetic clusters K ranging from 1 to 10 and the optimal number of clusters was chosen based on the cross-entropy of the best run for each value of K^33^. We tested the species status of the five clusters that did not correspond to already recognized species using Bayes factor delimitation^36^. This method relies on comparing the marginal likelihood of alternative species delimitation scenarios using under the multispecies coalescent model to identify evolutionary lineages that do not exchange genes anymore, *i.e.* species under the general species concept^34,35^. For each genetic cluster, alternative species delimitation scenarios in which the cluster would have species rank or not were statistically compared (Fig. S10). We note that for *A. pubescens* only four individuals were included in species delimitation analyses, which is less than generally recommended for SNAPP to perform well (i.e., less than five samples per species^36,38^).

The geographic distribution and bedrock affinities of all species were determined thanks to field data collected during our own sampling campaigns, in combination to data obtained from an online citizen-science project dedicated to the digitization and geo-referencing of herbarium records of the genus *Androsace* (http://lesherbonautes.mnhn.fr/missions/13798338). We also scrutinized 139 plant samples and coded a number of morphological characters, in order to provide morphological diagnosis criteria to differentiate study taxa. Once we had confirmed that these six taxa deserved species status, we inferred their phylogenetic relationships using species tree inference from unlinked SNPs with the program SNAPP^38^. Four independent chains of 500,000 steps were combined to produce a maximum clade credibility tree, which was calibrated to absolute time using the estimation of the crown age of /Helvetica obtained above. Finally, we tested possible hybrid origins of the three newly described species from *A. alpina* and *A. pubescens* as parent species using the diyABC software^37^ with 5 million simulations in each case.

## Taxonomic treatment: description of three novel *Androsace* species

### *Androsace delphinensis* Dentant, Lavergne, F.C. Boucher & S. Ibanez sp. nov

[= *Androsace pubescens* auct. non DC.]

*Holotypus (designated here*): France, Hautes-Alpes, Ecrins, Pic Coolidge (3775 m a.s.l.), close to the summit, in south-facing crevices in granite, [6,358341°/44,90947°], 3758 m a.s.l., 2019/08/13. Coll.: Dentant. GRM[MHNGr.2020.46703]!

*Etymology*: Named after the region of Dauphiné, in the southern French Alps.

Species description

Perennial plant forming a creeping cushion, sometimes compact, up to 5(10) cm high, 3–20 cm in diameter, made of loose to slightly compact rosettes. Leaves lanceolate, 7–10 × 1–1.5 mm, hairy on both sides. *Hairs* persistent, *simple* [proportion (0)25% to 75(100)%] *bifurcated* or *branched* (at least 3 branches), slightly curved, 0.2–0.5 mm long, composed of (3)4–6(8) cells. Hairs often branched with a short branch at the top, sometimes broken. *Pedicel with hairs exclusively bifurcated or branched*. *Corolla always white,* 7–8 mm in diameter. Flowering: June to August. Habitat: rock crevices on gneiss, granite, sandstone, flysch. Elevation: 2400 m to 3850 m a.s.l.. Chorology: Southwestern Alps (Écrins, Grandes Rousses, Belledonne).

### *Androsace vesulensis* Dentant, Lavergne, F.C. Boucher & S. Ibanez sp. nov

[= *Androsace pubescens* auct. non DC.; = *Androsace alpina* auct. non Lam.]

*Holotypus* *(designated here):* Italy, Piedmont, Monte Viso (3841 m a.s.l.), close to the summit from the normal route, in south-facing crevices in ophiolite, [7,08978°/44,66756°], 3750 m a.s.l., 2017/07/18. Coll.: Lavergne & Smyčka. GRM[MHNGr.2020.46704]!

*Etymology*: Named after Monte Viso, in the southwestern Italian Alps.

Species description

Perennial plant forming a creeping cushion, sometimes compact, up to 5(8) cm high, 3–10 cm in diameter, made of loose to slightly compact rosettes. Leaves lanceolate, 5–6.3 × 1–2.2 mm, hairy (mainly on edges). Hairs persistent, *deer-antler-shaped*, 0.1–0.2 mm long, composed of 3–4(7) cells. *Corolla always white*, 7 mm in diameter. Flowering: June to August. Habitat: rock crevices on ophiolite (basalt, gabbro, serpentine). *Elevation*: 2800 m to 3800 m a.s.l.. Chorology: endemic of Monte Viso and neighboring ophiolite summits (Italy and France).

### *Androsace saussurei* Dentant, Lavergne, F.C. Boucher & S. Ibanez sp. nov

[= *Androsace pubescens* auct. non DC.; = *Androsace alpina* auct. non Lam.; = *Androsace albimontana* D. Jord. & Jacquemoud ?].

*Holotypus (designated here)*: France, Haute-Savoie, Mont Blanc (4810 m a.s.l.), southern ridge of the *Rocher de l’Heureux Retour*, in crevices on granite [6,857218°/45,858224°], 3500 m a.s.l., 2020/06/03. Coll.: Lavergne, Bartalucci, Carlson & Dentant. GRM[MHNGr.2020.46705]!

*Etymology*: Named after the Genevan scholar Horace-Bénédict de Saussure (1740–1799), first promoter of science in high mountains.

Species description:

Perennial plant forming a creeping cushion, sometimes compact, up to 5(8) cm high, 3–10 cm in diameter, made of loose to slightly compact rosettes, 4.7–8.1 mm in diameter. Leaves lanceolate, 4.5–6(9.5) × 1.1–1.7 mm, hairy on both surfaces, *often reddish at the tips* (higher anthocyanin concentration). *Hairs* persistent, *simple* [proportion (0)25% to 75(100)%], *bifurcated* or *branched* (at least 3 branches), 0.2–0.5 mm long, composed of 3–5(8) cells. Pedicel with hairs exclusively bifurcated or branched. *Corolla white to purplish*, 7 mm in diameter. *Buds often purplish*. Flowering: June to August. Habitat: rock crevices on protogine and granite. *Elevation*: 2000 m to 4070 m a.s.l.—(highest elevation known for a vascular plant in Italy, observed by Brad Carlson in the south face of Mont Blanc, Italy). Chorology: Western Alps (Mont-Blanc, Gran Paradiso, Valais, Vanoise, Thabor).

## Supplementary Information

Supplementary Information 1.

Supplementary Information 2.
